# Prophylactic Ligation of the Innominate Artery and Creation of Tracheostomy in a Neurologically Impaired Girl: A Case Report

**DOI:** 10.1155/2011/790746

**Published:** 2011-10-05

**Authors:** Masayuki Obatake, Takayuki Tokunaga, Koji Hashizume, Kyoko Mochizuki, Takeshi Nagayasu

**Affiliations:** ^1^Division of Pediatric Surgery, Department of Surgical Oncology, Nagasaki University Graduate School of Biomedical Sciences, Nagasaki 852-8501, Japan; ^2^Department of Cardiovascular Surgery, Nagasaki University Graduate School of Biomedical Sciences, Nagasaki 852-8501, Japan; ^3^Division of Pediatric Surgery, Department of Surgery, Nagasaki University Graduate School of Biomedical Sciences, Nagasaki 852-8501, Japan

## Abstract

Tracheoinnominate artery fistula is known as a potentially fatal complication for patients who depend on tracheostomy or tracheoesophageal diversion. Since the bleeding from a TIF is often difficult to control, preventative procedures are recommended to avoid this complication. An 11-year-old girl with hypoxic-ischemic encephalopathy and scoliosis developed tracheal stenosis caused by compression from the innominate artery. Respiratory control with intubation through the tracheal stenosis was needed, and the patient was at high risk for developing a TIF. She underwent ligation of the innominate artery at tracheostomy. Subsequent tracheostomy revealed a widened tracheal lumen and no further complications. Prophylactic ligation of the innominate artery and creation of tracheostomy might be considered as a valid option for patients at high risk of developing TIF.

## 1. Introduction

Neurologically impaired children often require tracheostomy or tracheoesophageal diversion for long-term mechanical ventilation. Under these circumstances, numerous complications such as tracheal wall damage, tracheal granulation, and tracheoinnominate fistula (TIF) have been reported [1–3]. TIF is a serious and often fatal complication. Since the survival rate is reported to be less than 30% even with surgical intervention [4, 5], preventive measures are recommended to avoid TIF. We report a case of ligation of the innominate artery before tracheostomy in a patient who had developed tracheal stenosis.

## 2. Case Report

**Figure 1:**
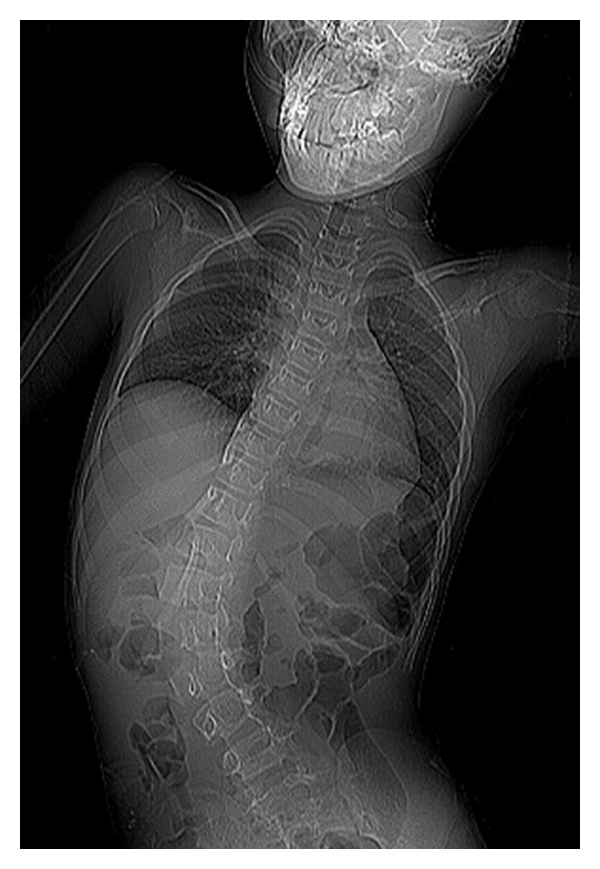
Chest X-ray image shows severe scoliosis.

**Figure 2:**
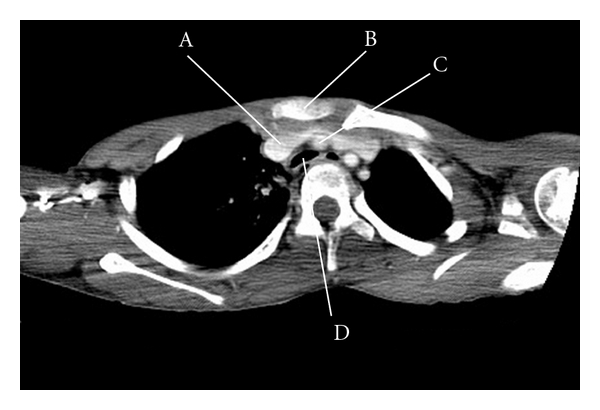
Chest computed tomography. Computed tomogram before the operation shows extrinsic compression of the trachea by the innominate artery. (A) Superior vena cava; (B) manubrium; (C) innominate artery; (D) trachea.

**Figure 3:**
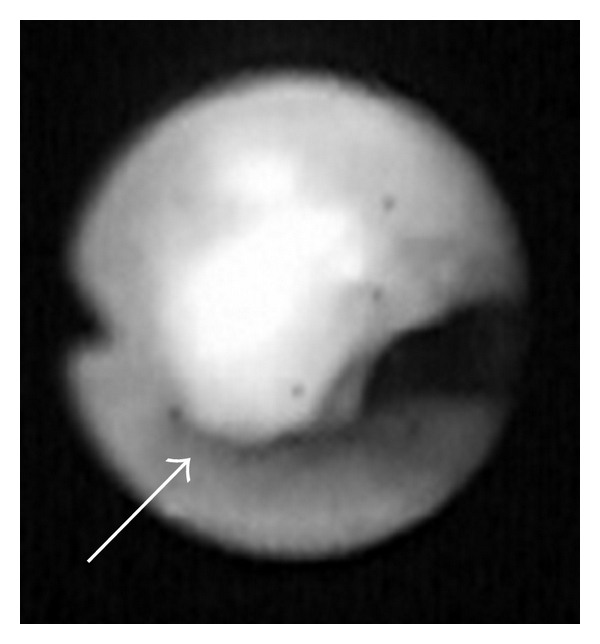
Bronchoscopy. Bronchoscopy shows narrowing of the trachea by a pulsatile compression on the anterior tracheal wall (white arrow).

An 11-year-old girl with hypoxic-ischemic encephalopathy had gradually developed scoliosis, and she had experienced several episodes of mild wheezing associated with myotonia. Her SpO_2_ levels had decreased to 20–30% during episodes of severe myotonia. She was referred to a medical center after the abrupt onset of wheezing and respiratory distress. Chest X-ray revealed scoliosis (Figure 1), and laryngoscopy showed neither supraglottic stenosis nor edema. Chest computed tomography (CT) revealed tracheal stenosis caused by compression by the innominate artery (Figure 2). Bronchoscopy demonstrated narrowing of the trachea by external compression of its anterior wall as well as tracheomalacia (Figure 3) involving the middle trachea to the tracheal bifurcation. An uncuffed endotracheal tube was inserted distally to the stenosis, and this resulted in a prompt improvement in her respiratory condition. However, she was thought to require long-term tracheal intubation because of the tracheal stenosis and tracheomalacia. Furthermore, she was considered at increased risk of developing tracheal damage and subsequent formation of TIF from tracheal intubation. She was transported to our hospital for preventative ligation of the innominate artery and tracheostomy. 

**Figure 4:**
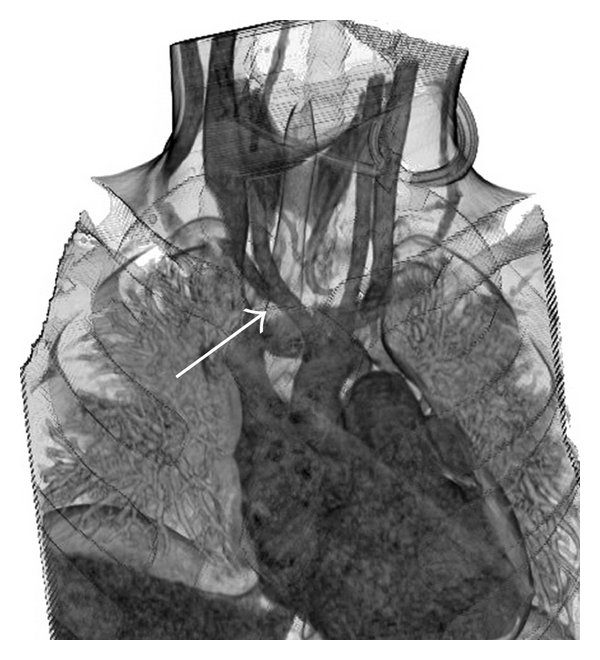
3D CT. Three-dimensional CT shows the innominate artery (white arrow) crossing the trachea from left to right just cranial to the manubrium.

Brain magnetic resonance CT showed a normal circle of Willis with no occlusion of the internal carotid or innominate arteries. 3D CT demonstrated that the innominate artery crossed the trachea from left to right just cranial to the manubrium (Figure 4). We planned prophylactic ligation of the innominate artery to prevent the formation of TIF.

**Figure 5:**
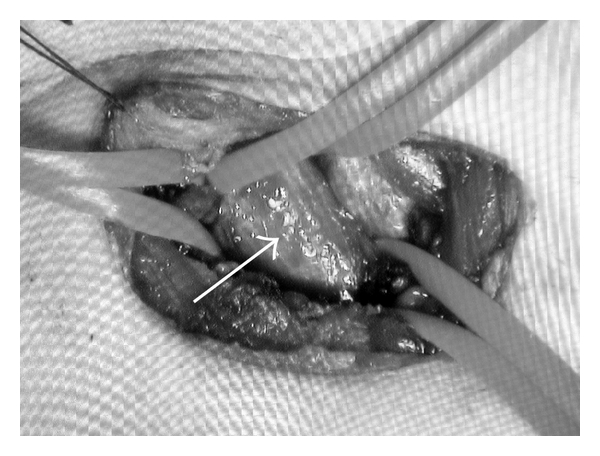
Intraoperative view. The innominate artery (white arrow) was isolated and taped proximally and distally.

The patient was positioned supine under general anesthesia. A small curvilinear transverse skin incision was made just above the suprasternal notch. The innominate artery was exposed anterior to the trachea (Figure 5). After clamping of the innominate artery, the SpO_2_, as measured at the right hand and oxygen saturation (rSO_2_) in both cerebral hemispheres, was initially reduced to 70% of baseline readings, but promptly increased to 90%. The innominate artery was transected, and both stumps were closed with continuous absorbable sutures. Thymus tissue was interposed between the proximal and distal stump. After tracheostomy at the 3rd tracheal ring, bronchoscopy revealed a widened tracheal lumen. She was transferred to the intensive care unit for postoperative recovery. The postoperative course was eventful with no neurological complications, and she was discharged on the 6th postoperative day. 

## 3. Discussion

TIF is an uncommon complication, affecting fewer than 1% of patients after tracheostomy or tracheoesophageal diversion and occurring within 2-3 weeks of these procedures [6–8]. Although the mechanism of TIF is not clear, several factors are implicated, including pressure on the trachea from the adjacent innominate artery, which is exacerbated by scoliosis and pressure necrosis of the tracheal wall from the tracheostomy tube [3, 8, 9]. Progressive scoliosis induces narrowing of the mediastinum that results in the compression of the innominate artery against the trachea [10, 11]. 

Since hemorrhage from TIF is often difficult to control and is frequently fatal, preventive procedures are recommended to avoid this complication. Low tracheostomy placement (below the 4th tracheal ring) and excessive cuff pressure should be avoided [3, 8, 9]. Modern soft, flexible tracheostomy tubes are useful to prevent excessive pressure on the tracheal wall [3]. 

Once TIF has developed, successful management depends on early diagnosis and management. Immediate management includes minimizing blood loss using digital pressure and overinflation of the tracheostomy cuff over the bleeding point after securing the airway [8, 9, 12, 13]. In general, neurologically impaired patients are smaller than those who are neurologically intact, and digital pressure control of bleeding is quite difficult because of the small tracheotomy opening. Definitive control of hemorrhage requires surgical intervention or an endovascular technique to access the bleeding site. With recent developments in endovascular instruments, endovascular stent graft treatment of TIF has been reported [14]. 

The survival rate of TIF is reported to be less than 30%, even with surgical intervention [4, 11]. Periodic tracheoscopy is therefore important to detect early signs of TIF, particularly for cases with herald bleeding from the tracheostomy, presenting as hemoptysis or blood on suction [9]. A definitive indication for surgical treatment is bleeding from TIF, and most surgical interventions are performed after TIF formation. Prophylactic surgical intervention for TIF has been described less frequently. Iodice et al. reported prophylactic ligation of the innominate artery for 7 patients with neuromuscular disorders who had already been tracheostomized [3]. Tanaka and Iwanaka reported one neurologically impaired patient who underwent simultaneous ligation of the innominate artery and tracheoesophageal diversion [15]. Prophylactic preoperative and postoperative antibiotics were used to prevent mediastinitis. We consider that the present case had a high risk of TIF formation because the innominate artery was already encroaching on the trachea, hence long-term intubation was anticipated. The intubation tube should be placed beyond the encroaching innominate artery because of tracheal stenosis. In these circumstances, the encroaching innominate artery rubbed by the intubation tube had risk of developing TIF. A rate of future TIF formation for this patient without the ligation of innominate artery is not anticipated, but might be higher than a patient of no encroaching innominate artery in the trachea lumen. Allan and Wright reported the safety and reliability of ligation of the innominate artery [16]. Despite the reverse flow in the right internal carotid artery after ligation of the innominate artery, symptoms of subclavian steal have not been reported. Many of ligations of the innominate artery were performed as emergency procedures, and no time for checking an appearance of a circle of Willis. Preoperative examination of a circle of Willis is a prerequisite for the case of planning prophylactic ligation of the innominate artery. In the case of an occlusion of circle of Willis, revascularization to preserve the right carotid and right subclavian artery flow should be considered. 

We do not propose ligation of the innominate artery as a routine prophylactic procedure at tracheostomy for all neurologically impaired patients. The treatment of TIF under emergency conditions was not really successful, and we believe that prophylactic ligation is a valid procedure for patients at high risk of developing TIF and helped to restore the patient's condition.

## 4. Conclusion

Although prophylactic ligation of the innominate artery might be the most effective and least invasive surgical intervention to prevent TIF, it is not a routine procedure for patients with tracheostomy and tracheoesophageal diversion. Nonetheless, it might be considered a valid option for patients at high risk of developing TIF.

## References

[1] Kapural L, Sprung J, Gluncic I (1999). Tracheo-innominate artery fistula after tracheostomy. *Anesthesia and Analgesia*.

[2] Ross CB, Morris JA (1988). Tracheo-innominate artery fistula: a potentially fatal complication of tracheostomy. *Journal of the Tennessee Medical Association*.

[3] Iodice F, Brancaccio G, Lauri A, Di Donato R (2007). Preventive ligation of the innominate artery in patients with neuromuscular disorders. *European Journal of Cardiothoracic Surgery*.

[4] Schaefer OP, Irwin RS (1995). Tracheoarterial fistula: an unusual complication of tracheostomy. *Journal of Intensive Care Medicine*.

[5] Takasaki K, Enatsu K, Nakayama M, Uchida T, Takahashi H (2005). A case with tracheo-innominate artery fistula: successful management of endovascular embolization of innominate artery. *Auris Nasus Larynx*.

[6] Allan JS, Wright CD (2003). Tracheoinnominate fistula: diagnosis and management. *Chest Surgery Clinics of North America*.

[7] Grillo HC, Donahue DM, Mathisen DJ, Wain JC, Wright CD (1995). Postintubation tracheal stenosis: treatment and results. *Journal of Thoracic and Cardiovascular Surgery*.

[8] Hung JJ, Hsu HS, Huang CS, Yang KY (2007). Tracheoesophageal fistula and tracheo-subclavian artery fistula after tracheostomy. *European Journal of Cardiothoracic Surgery*.

[9] Silva RC, Chi DH (2010). Successful management of a tracheo-innominate fistula in a 7-year-old child. *International Journal of Pediatric Otorhinolaryngology*.

[10] Saito T, Sawabata N, Matsumura T, Nozaki S, Fujimura H, Shinno S (2006). Tracheo-arterial fistula in tracheostomy patients with Duchenne muscular dystrophy. *Brain and Development*.

[11] Hasegawa T, Zaima A, Hisamatsu C, Nishijima E, Okita Y (2010). Minimally invasive innominate artery transection for tracheomalacia using 3-dimensional multidetector-row computed tomographic angiography: report of a case. *Journal of Pediatric Surgery*.

[12] Ailawadi G (2009). Technique for managing tracheo-innominate artery fistula. *Operative Techniques in Thoracic and Cardiovascular Surgery*.

[13] Singh N, Fung A, Cole IE (2007). Innominate artery hemorrhage following tracheostomy. *Otolaryngology—Head and Neck Surgery*.

[14] Deguchi JO, Furuya T, Tanaka N (2001). Successful management of tracheo-innominate artery fistula with endovascular stent graft repair. *Journal of Vascular Surgery*.

[15] Tanaka Y, Iwanaka T (2009). Ligation of the innominate artery at tracheoesophageal diversion in neurologically impaired children for preventing trachea-innominate artery fistula. *Japanese Journal of Pediatric Surgery*.

[16] Allan JS, Wright CD (2003). Tracheoinnominate fistula: diagnosis and management. *Chest Surgery Clinics of North America*.

